# Supplementary material for: Strainer-Separable TiO2 on Halloysite Nanocomposite-Embedded Alginate Capsules with Enhanced Photocatalytic Activity for Degradation of Organic Dyes
Source: Nanomaterials (Basel). 2022 Jul 10;12(14):2361. doi: 10.3390/nano12142361 (PMC9320041; doi:10.3390/nano12142361)
Supplement: Supplementary file 1 [file nanomaterials-12-02361-s001.zip › nanomaterials-1807670-supplementary.pdf]

Supplementary Materials

Strainer-separable TiO_2 on halloysite nanocomposite-embedded alginate capsules with enhanced photocatalytic activity for degradation of organic dyes

Jewon Lee[1], Sicheon Seong[2], Soyeong Jin[2,3], Jaeyong Kim[4], Youngdo Jeong[3,5,*], and Jaegeun Noh[1,2,6,*]

[1] Department of Convergence of Nanoscience, Hanyang University, 222 Wangsimni-ro, Seongdong-gu, Seoul 04763, Korea; chu0254@naver.com (J.L.)

[2] Department of Chemistry, Hanyang University, 222 Wangsimni-ro, Seongdong-gu, Seoul 04763, Korea; ssc09122@hanyang.ac.kr (S.S.); truejin@hanyang.ac.kr (S.J.), jgnoh@hanyang.ac.kr (J.N.)

[3] Center for Biomaterials, Biomedical Research Institute, Korea Institute of Science and Technology (KIST), Seoul, 02792, Korea; zerodegree@kist.re.kr (Y.J.)

[4] Department of Physics, Hanyang University, 222 Wangsimni-ro, Seongdong-gu, Seoul 04763, Korea; kimjy@hanyang.ac.kr (J.K)

[5] Department of HY-KIST Bio-convergence, Hanyang University, 222 Wangsimni-ro, Seongdong-gu, Seoul 04763, Republic of Korea

[6] Institute of Nano Science and Technology, Hanyang University, 222 Wangsimni-ro, Seongdong-gu, Seoul 04763, Korea

*Correspondence: zerodegree@kist.re.kr (Y.J.); jgnoh@hanyang.ac.kr (J.N.)

1. Chemicals and materials

Halloysite nanotubes (HNTs) were purchased from Sigma Aldrich (30–70 nm × 1–3 μm , nanotube, Cat. #: 1332-58-7). Sodium alginate (98%, Cat. # 13035-1201), titanium isopropoxide (98%, Cat. #:45040-0430, TTIP), and rhodamine B (Cat. #: 87180-1510, Chemical pure grade) were purchased from Junsei Chemical Co., Ltd. Calcium chloride anhydrous (93%, Cat. #: 2507-1405) and isopropyl alcohol were purchased from Daejung Chemicals & Metals Co. Ltd. Nitric acid (60%, Cat. #:005N0233) was purchased from Samchun Pure Chemical Co., Ltd.

2. Preparation of TiO2@HNTs composite

In brief, 5 mL of TTIP was added in a 200 mL round-bottom flask containing 24 mL of isopropanol. A mixture of 16 mL of water, 16 mL of isopropanol, and 0.2 mL of nitric acid were added dropwise to the round-bottom flask containing the reaction mixture, with vigorous stirring. After 2 h stirring, 1.3 g of HNT was added, with 2 h stirring. Through 1-day aging at room temperature, the TiO_2@HNTs were formed. Through centrifugation (8000 rpm for 10 minutes) and several washing steps with ethanol/water mixture (1:1), the TiO_2@HNTs were purified. To gain the powder form of catalysts, the samples were dried in a vacuum oven at 85 °C for 12 h and then were ground and calcined at 350 °C for 2 h (2 °C /min rates) under ambient conditions.

3. Structural analysis of TiO_2@HNT/Alcaps

The morphology of capsules was analyzed using a scanning field emission electron microscope (FE-SEM, Hitachi, s-4800 instrument). To examine the thermal degradation characteristics of alginate gel and TiO_2@HNTs/Alcaps, thermogravimetric analysis (TGA) was performed using the SDT Q600, TA Instruments (Heating: 10 °C/min, N_2 = 100 mL/min, 800 °C). UV-vis measurements were performed with Evolution 60S (Thermo Fisher Scientific).

4. Measurement of rhodamine B adsorption

The dye adsorption experiments were evaluated by adding 0.1 g of alginate, HNTs/Alcap, TiO_2/Alcaps, and TiO_2@HNTs/Alcaps to 20 ml of 5.0 mg/L rhodamine B solution. The absorbance at 554 nm of the rhodamine B solution was monitored under stirring (800 rpm) conditions for 1 h. To calculate the removal efficiency (%), q_t (the rhodamine B adsorption capacity, unit: mg/g), q_e (the rhodamine B adsorption capacity at equilibrium, unit: mg/g), and k_2 (rate constant, unit: g/mg min), we used the following the equations:

$$\text{Removal efficiency (\%)} = \frac{C_0 - C_e}{C_0} \times 100 \quad (1)$$

$$q_e\ (\text{mg/g}) = \frac{(C_0 - C_e)V}{m} \quad (2)$$

$$q_t\ (\text{mg/g}) = \frac{(C_0 - C_t)V}{m} \quad (3)$$

$$\frac{t}{q_t} = \frac{1}{k_2 q_e^2} + \frac{1}{q_e} t \quad (4)$$

where C_0 and C_t are the concentrations of dye at initial and t time (unit: mg/L), respectively; m and V are the weight of the catalyst (unit: g) and the volume of dye solution (unit: L), respectively.

5. Photodegradation of rhodamine B using the capsules

For the photocatalytic dye degradation of rhodamine B, 0.1 g of alginate, HNTs/Alcaps, TiO_2/Alcaps, and TiO_2@HNT/Alcaps were added to 20 mL of 5.0 mg/L rhodamine B solution, respectively. The solutions were stirred in the dark for 1 h. Then, we applied UV light (250 W, 356 nm) using a lamp (Ushio-SP9) to monitor the change of absorbance induced by the dye degradation. The dye degradation efficiency (%) and the rate constant were obtained using the equations below [33].

$$\text{Degradation efficiency (\%)} = \frac{C_0 - C}{C_0} \times 100 \quad (5)$$

$$\ln \frac{C_0}{C} = k_1 t \quad (6)$$

where C_0 and C are the dye concentrations at initial and final times (unit: mg/L) and k_1 is the rate constant of the pseudo first order (unit: min^{-1}).

6. Instruments

6.1. TEM

TEM samples were prepared by placing one drop of the desired catalyst solution (~1–3 μM) onto a 300-mesh copper grid coated with carbon film, followed by drying. The samples were analyzed and photographed using a JEM 2100F (JEOL) transmission electron microscope at an accelerating voltage of 80 kV.

6.2. SEM

FE-SEM samples were prepared by placing one drop of the desired catalyst solution (~1–3 μM) onto a silicon wafer. After drying, we sputter-coated the sample substrate with platinum for 60 s using an MC1000 Ion sputter coater (Hitachi High-Tech). The samples were analyzed and photographed using a Hitachi S-4800 scanning electron microscope with an accelerating voltage of 5 kV.

Supporting Figures

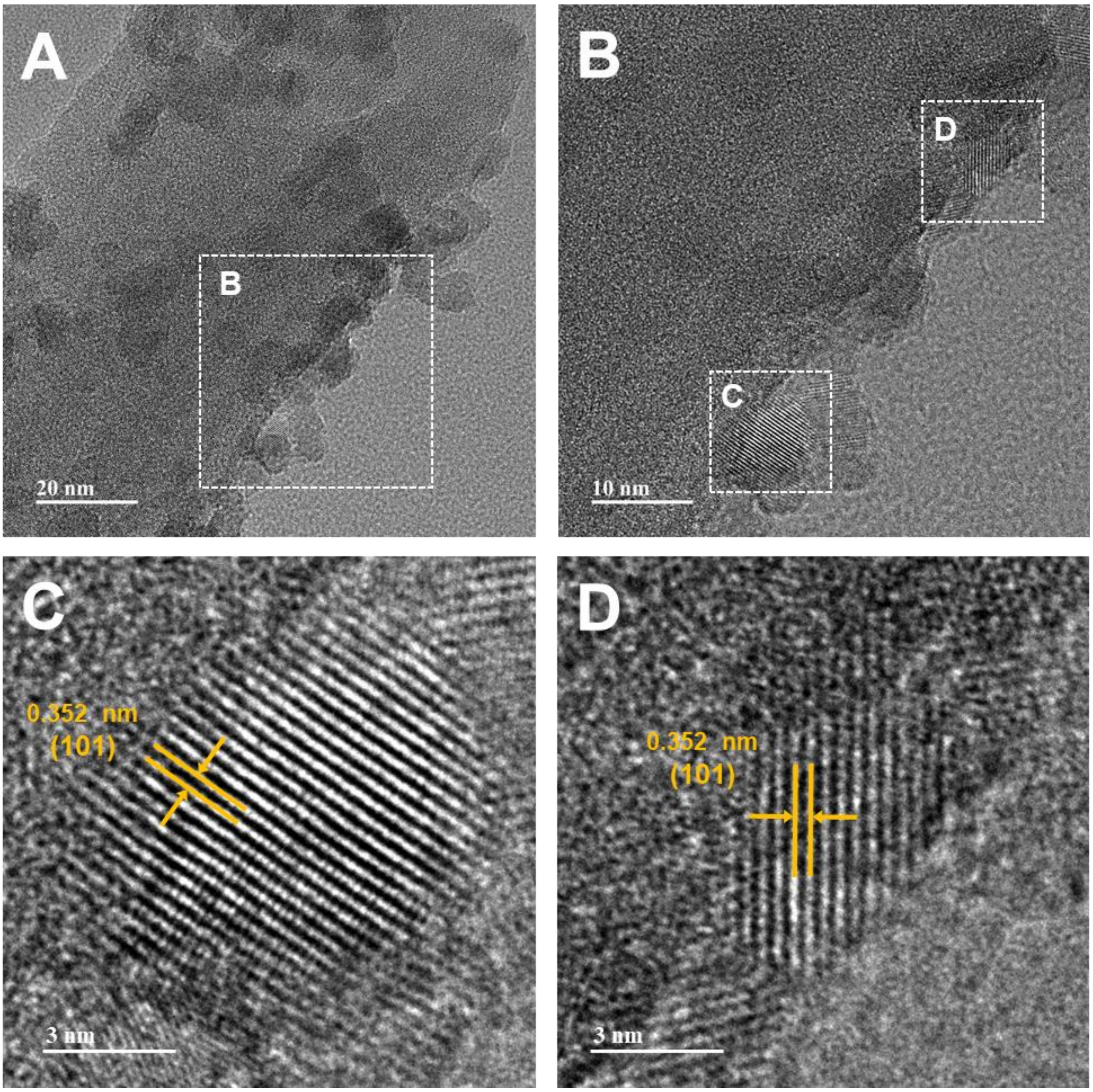

Figure S1. High-resolution TEM images of TiO_2@HNT nanocomposites.

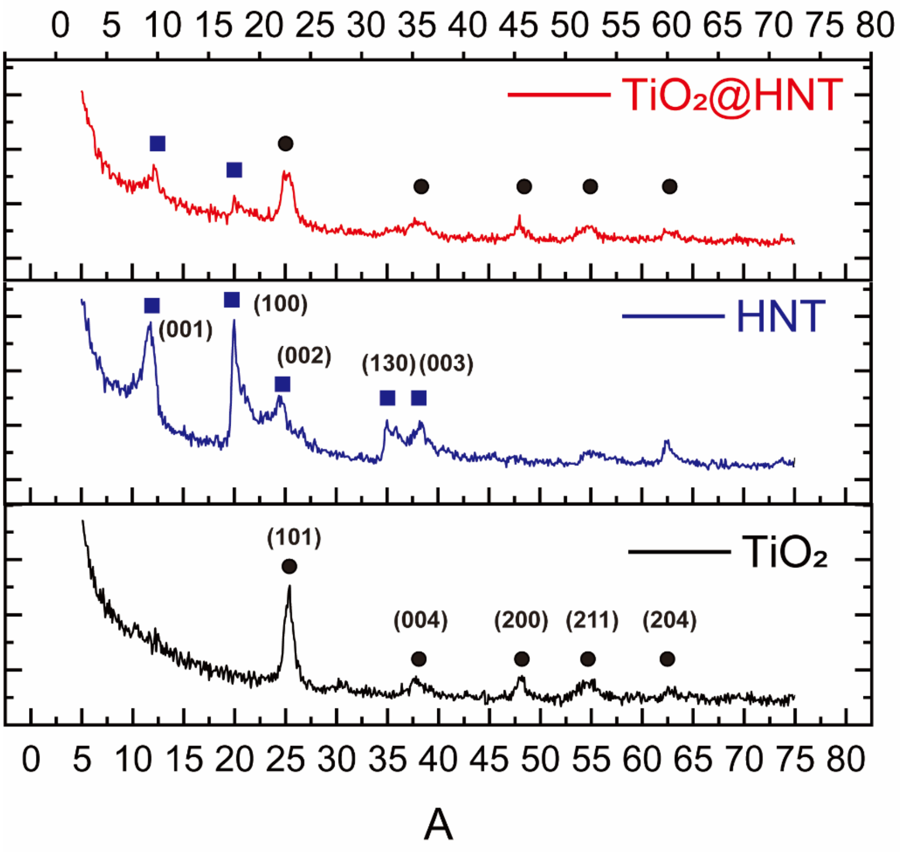

Figure S2. XRD patterns of TiO_2, HNTs, and TiO_2@HNTs.

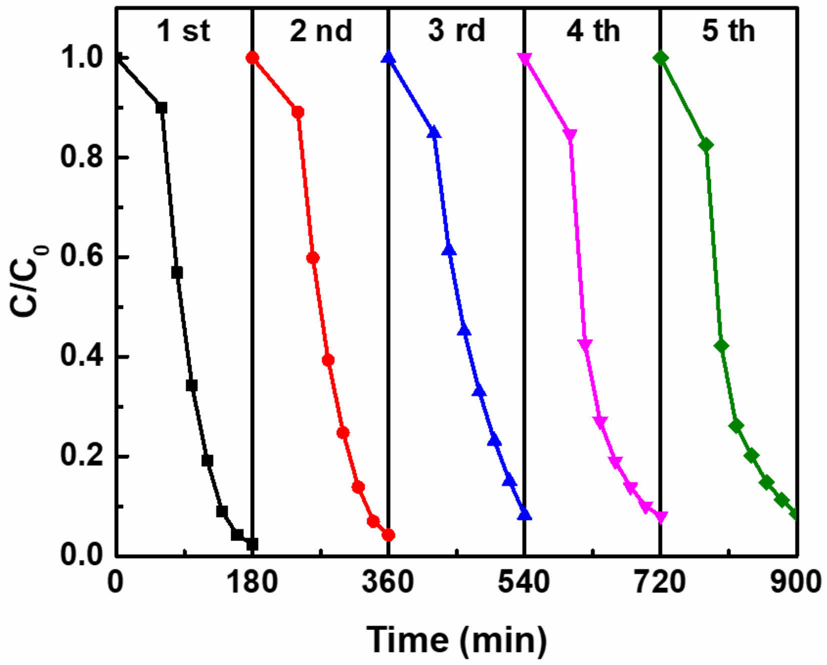

Figure S3. Catalytically recyclable degradation of dye with TiO_2@HNT/Alcaps.

Table S1. Pseudo-second-order kinetic model parameters of rhodamine B adsorption by catalyst composite capsules.

Sample	q_e (mg/g)	k_2 (g/mg min)	R^2
**Adsorption**			
Alginate	0.1026	1.9690	0.9967
HNT/Alcap	0.1191	1.2501	0.9961
TiO_2/Alcap	0.1420	0.7619	0.9948
TiO_2@HNT/Alcap	0.1704	0.5635	0.9932

Table S2. Pseudo-first-order kinetic parameters of photocatalytic degradation of rhodamine B by catalyst composite capsules.

Sample	Dye removal %	k_1 (min^{-1})	R^2
**UV irradiation**			
Dye	9.980	0.0007	0.9918
Alginate gel	20.30	0.0017	0.9963
HNT/Alcap	38.01	0.0031	0.9943
TiO_2/Alcap	51.32	0.0052	0.9933
TiO_2@HNT/Alcap	97.65	0.0312	0.9939
